# Editorial: Advanced (gene and cell) therapies for central nervous system applications

**DOI:** 10.3389/fnmol.2023.1140949

**Published:** 2023-02-01

**Authors:** Liliana Mendonça, Christopher Webster, Johannes Boltze, Clévio Nóbrega

**Affiliations:** ^1^Center for Neuroscience and Cell Biology (CNC), University of Coimbra, Coimbra, Portugal; ^2^Institute of Interdisciplinary Research, University of Coimbra, Coimbra, Portugal; ^3^Faculty of Science and Technology, University of Algarve, Faro, Portugal; ^4^Department of Neuroscience, Sheffield Institute for Translational Neuroscience (SITraN), University of Sheffield, Sheffield, United Kingdom; ^5^Neuroscience Institute, University of Sheffield, Sheffield, United Kingdom; ^6^School of Life Sciences, University of Warwick, Coventry, United Kingdom; ^7^Algarve Biomedical Center Research Institute (ABC-RI), University of Algarve, Faro, Portugal; ^8^Faculty of Medicine and Biomedical Sciences, University of Algarve, Faro, Portugal

**Keywords:** gene augmentation, gene silencing, gene editing, viral and non-viral vectors for gene therapy, cell therapy, cell replacement and neuroprotection, brain experimental models-brain organoids, brain delivery and brain diseases

Advanced therapies as defined by the European Medicines Agency (EMA) comprises strategies involving gene therapy, cell therapy, and tissue engineering. Overall, these strategies offer a wide range of possibilities to treat and cure diseases, including those affecting the central nervous system (CNS). In this line, *Advanced (Gene and Cell) Therapies for Central Nervous System Applications* Research Topic was intended to provide a platform for researchers to publish their findings, contributing to the continuous advance of this research area.

Nine papers were accepted and published in this Research Topic, from which four described original research data, two papers were reviews and two mini reviews, and one paper focused on hypothesis and theory.

In the paper *Administration of Variants AAV-PHP.B and AAV-PHP.eB on Brain Transduction in Adult Rhesus Macaques* (Arotcarena et al.), presented new data on the biodistribution and CNS transduction efficiencies after lumbar intrathecal bolus delivery of identical doses of either AAV-PH.B:CAG-EGFP or AAV-PHP.eB:CAG-EGFP in rhesus macaque monkeys. This proof-of-concept work provides valuable data on the use of AAV-PHP.B and AAV-PHP.eB to distribute genetic material in the CNS with cell-type specificity, contributing to the advance of gene therapy strategies targeting the CNS.

Deng et al., in their paper *An in vivo Cell-Based Delivery Platform for Zinc Finger Artificial Transcription Factors in Pre-clinical Animal Models* presented a mesenchymal stem/stromal cell (MSC)-based delivery system for the secretion of a zinc finger (ZF) protein and assessed it regarding efficacy and safety in mice and rhesus monkeys, respectively. It was shown that secreted ZF ameliorated motor deficits in an Ube3a deletion Angelman Syndrome (AS) mouse model. Intrathecally administered autologous rhesus MSCs were well tolerated and ZF protein was detected within the cerebrospinal fluid, midbrain, and spinal cord. Therefore, this study successfully provides new data for the functionality of an MSC-based secretion platform for the delivery of gene modifying artificial transcription factors such as ZF. It is also of translational interest.

The article *Expression and Prognostic Role of Glia Maturation Factor-*γ *in Gliomas* by Liu et al. investigated the role of glia maturation factor-γ (GMFG) in gliomas. Public datasets comprising 2,518 gliomas samples and an in-house cohort containing 120 gliomas samples were evaluated. Bioinformatic tools were used to assess the correlation between GMFG expression and immune cell infiltration and to determine the prognostic role of GMFG and its association with temozolomide (TMZ) response in gliomas. GMFG expression was found higher in gliomas and was associated with a bad response to TMZ treatment. This work supports GMFG as a prognostic biomarker for low-grade glioma and glioblastoma multiforme and describes important progress in the development of advanced therapies for gliomas.

*Expression and potential role of FOSB in glioma* by Qi et al. addresses the expression and biological role of FOSB in glioblastoma multiforme. Authors found that FOSB expression was higher in glioma compared with normal brain tissue and that FOSB downregulation through lentiviral vector delivery of shRNA FOSB led to a decrease in glioma cells viability, as well as their ability to proliferate and migrate. It was also shown that down-regulation of FOSB decreases the growth of glioma cells transplanted to nude mice. Therefore, this study advances the targeting of FOSB for gene therapy approaches for this type of tumors.

In the review article *Treating Metastatic Brain Cancers With Stem Cells* (Sadanandan et al.), considered the immunological and inflammatory responses associated with Blood-Brain Barrier (BBB) damage secondary to cancer and the involvement of this pathological process in the growth and formation of metastatic brain cancers. Moreover, the use of bone marrow-derived stem cells to regenerate impaired endothelial cells of the BBB to attenuate brain metastasis was reviewed. The paper provided a critical discussion of the current advantages and disadvantages of stem cell-based therapy for metastatic brain cancer treatment.

In *Current Status of Mesenchymal Stem/Stromal Cells for Treatment of Neurological Diseases* (Soares et al.) reviewed the current knowledge of the therapeutic potential of MSC-based therapies for neurological diseases. The paper also focuses on the challenges of culture conditions, quality control, and the development of potency tests. This review paper can serve as a compendium for researchers being interested in more efficient ways to generate future cell therapy products.

The mini review *The Effect of miRNA-Modified Exosomes in Animal Models of Spinal Cord Injury: A meta-Analysis* by Hu et al., described a meta-analysis focused on evaluating the overall efficacy of therapies based on miRNA-modified exosomes, namely miR-133 and miR-26, on functional recovery in animal models of spinal cord injury (SCI). From the 11 preclinical studies included in the paper, authors conclude that miRNA-modified exosomes have shown great potential in the treatment of SCI, providing important insights on the potential use of exosomes as gene delivery vehicles for SCI treatment.

Xiaoshuai et al. in *Advantages of CRISPR-Cas9 combined organoid model in the study of congenital nervous system malformations* reviewed and discussed congenital nervous system malformations (CNSM) as well as the different modeling methods available for these diseases. The use of organoids to model CNSM and the application of CRISPR-Cas9 in the organoid platform to study the pathogenesis and treatment strategies were also discussed. This mini-review highlighted the potential of CRISPR-Cas9 edited brain organoid technology to uncover the genetic complexity and disease pathogenesis of CNSM.

The Hypothesis and Theory article *Potential effects of commonly applied drugs on neural stem cell proliferation and viability: A hypothesis-generating systematic review and meta-analysis* by Mortimer et al. conducted a systematic review and meta-analysis looking at potential effects of commonly prescribed antihypertensive and antihyperlipidemic medication on NSC viability, proliferation, and differentiation. The meta-analysis revealed evidence that alpha-2 adrenoceptor agonists and various statins may have an inhibiting effect on NSC proliferation and that L-type calcium channel blockers and statins, particularly lovastatin, may reduce NSC viability. From these findings, authors provide insights into how future research on this topic may be conducted.

Overall, the articles in the present Research Topic advanced on the development of new gene delivery tools and vehicles and new targets and therapeutic strategies, such as cell-based strategies to treat incurable diseases.

## Author contributions

LM, CW, JB, and CN wrote and reviewed the manuscript and approved the submitted version.

